# Identification and validation of a miRNA-related expression signature for tumor mutational burden in colorectal cancer

**DOI:** 10.1186/s12957-021-02137-1

**Published:** 2021-02-20

**Authors:** Lijun Xu, Qing Zheng

**Affiliations:** Division of Gastroenterology and Hepatology, Key Laboratory of Gastroenterology and Hepatology, Inflammatory Bowel Disease Research Center, Shanghai Institute of Digestive Disease, Renji Hospital, School of Medicine Shanghai Jiao Tong University, Ministry of Health, 160# Pu Jian Ave, Shanghai, 200127 China

**Keywords:** Colorectal cancer, Immune checkpoint inhibitors, MicroRNAs, TCGA, Tumor mutational burden

## Abstract

**Background:**

Tumor mutational burden (TMB) is a promising predictor, which could stratify colorectal cancer (CRC) patients based on the response to immune checkpoint inhibitors (ICIs). MicroRNAs (miRNAs) act as the key regulators of anti-cancer immune response. However, the relationship between TMB and miRNA expression profiles is not elucidated in CRC.

**Methods:**

Differentially expressed miRNAs (DE miRNAs) between the TMB^high^ group and the TMB^low^ group were identified for the CRC cohort of the TCGA database. In the training cohort, a miRNA-related expression signature for predicting TMB level was developed by the least absolute shrinkage and selection operator (LASSO) method and tested with reference to its discrimination, calibration, and decision curve analysis (DCA) in the validation cohort. Functional enrichment analysis of these TMB-related miRNAs was performed. The correlation between this miRNA-related expression signature and three immune checkpoints was analyzed.

**Results:**

Twenty-one out of 43 DE miRNAs were identified as TMB-related miRNAs, which were used to develop a miRNA-related expression signature. This TMB-related miRNA signature demonstrated great discrimination (AUC_test set_ = 0.970), satisfactory calibration (*P* > 0.05), and clinical utility in the validation cohort. Functional enrichment results revealed that these TMB-related miRNAs were mainly involved in biological processes associated with immune response and signaling pathways related with cancer. This miRNA-related expression signature showed a median positive correlation with PD-L1 (R = 0.47, *P* < 0.05) and CTLA4 (*R* = 0.39, *P* < 0.05) and a low positive correlation with PD-1 (*R* = 0.16, *P* < 0.05).

**Conclusion:**

This study presents a miRNA-related expression signature which could stratify CRC patients with different TMB levels.

## Introduction

Colorectal cancer (CRC) is a commonly diagnosed cancer, and its incidence and mortality rate rank the third and second among all the malignant tumors, respectively [1]. With the increasing incidence of CRC in the young, there will be about 2.5 million newly diagnosed CRC cases in 2035 [2]. Although the current treatment strategy of surgical resection combined with radiotherapy and chemotherapy has extended survival time for early-stage CRC patients, poor prognosis still remains a serious problem for metastatic CRC [2, 3].

In recent years, several studies have identified that the interaction between programmed death receptor (PD-1) and its ligand, programmed death ligand (PD-L1), could serve as the mechanism for tumors to evade an antigen-specific T cell immunologic response [4]. Based on this hypothesis, immunotherapy is introduced and has revolutionized the approach to treatment for CRC [5]. Conventional chemotherapy kills tumor cells by interfering directly with DNA or targeting key proteins required for cell proliferation [6]. By contrast, the immunotherapy could induce cell death by restoring the dysfunctional antitumor T cells [7]. The most widely used immunotherapy in CRC is the immune checkpoint inhibitors (ICIs), which included PD-1/PD-L1 inhibitors and cytotoxic T-lymphocyte antigen 4 (CTLA-4) inhibitors. At present, PD-L1 expression by immunohistochemistry test has been used to identify CRC patients who can benefit from ICIs [8]. However, the PD-L1 expression could be regulated by the tumor microenvironment, and the correlation between PD-L1 expression and immunotherapy efficacy is not clear. Microsatellite instability (MSI) is also an established biomarker for predicting response to ICIs [9]. However, the response rate of ICIs is variable among CRC patients with high microsatellite instability (MSI-H), and responders have more somatic mutations and neoantigen loads than non-responders [10], indicating that additional predictive biomarkers are required.

Tumor mutational burden (TMB) is a promising independent predictor, which could stratify patients based on the response to ICIs [11, 12]. The definition of TMB is the number of somatic variants in the coding region of tumor genes. A recent study reveals that the response rate to ICIs in patients with TMB^high^ level is higher than that in patients with TMB^low^ level, suggesting that TMB^high^ level is positively correlated with immunotherapy efficacy [13]. However, TMB has not been widely used in clinical practice, mainly due to the non-standardization of TMB detection [14]. A large number of genetic mutations in TMB could produce “non-self” neoantigen proteins which could activate anti-tumor immune response [15, 16]. The post-transcriptional regulation is essential in the translation of these mutated genes into neoantigen proteins, and microRNAs (miRNAs) are involved in this process.

miRNA is one type of endogenous non-coding RNAs consisting of approximately 21–25 nucleotides that participates in the post-transcriptional modification process, and abnormal miRNA expression is involved in the pathogenesis of various types of cancer [17]. Some research has reported that miRNAs could serve as promising predictors for TMB levels and are involved in the regulation of anti-cancer immune response [18, 19]. For example, Lv et al. revealed that the expression profiles of miRNAs were related with TMB levels, and a miRNA-related signature classifier was developed to predict TMB level in lung adenocarcinoma [20]. Zhao et al. reported that miR-138-5p could bind to 3′ untranslated region (UTR) of immune checkpoint PD-L1, consequently leading to the inhibition of its translation [21]. However, the relationship between TMB and miRNA expression patterns is not elucidated in CRC. Thus, the purpose of this research lies in the development of a miRNA-related expression signature, which could identify CRC patients with different TMB levels.

## Materials and methods

### Data acquisition

Both somatic mutation data and miRNA expression profiles of the CRC cohort were downloaded from The Cancer Genome Atlas (TCGA) (https://portal.gdc.cancer.gov/), which provided comprehensive genomic information in various types of cancer. For somatic mutation data, the workflow type used in this research was VarScan2 Variant Aggregation and Masking. We measured the TMB of each sample based on the number of somatic mutations per DNA megabase [22]. Thirty-eight megabase was used to estimate the exome size [23]. We selected 10 mutations per megabase as a cutoff point, which separated CRC patients into TMB^high^ samples and TMB^low^ samples [24, 25]. The data type for miRNA expression profiles in our study was isoform expression quantification, which contained preprocessed mature miRNAs in 539 CRC samples and 9 control samples. A total of 457 samples with both somatic mutation data and miRNA expression profiles were extracted as the overall cohort. We then randomly assigned CRC samples in the overall cohort to either the training cohort (60%) or the validation cohort (40%).

### Identification of differentially expressed miRNAs

In the training cohort, we removed the miRNAs which contained missing values in more than 10% of the CRC samples. Differentially expressed miRNAs (DE miRNAs) between the TMB^high^ group and the TMB^low^ group were determined by the limma package in the R software. The |log2 fold change (FC)| > 1.5 and false discovery rate (FDR) < 0.01 were chosen as the threshold criteria. To visualize the expression patterns of these DE miRNAs, the heatmap was plotted using the pheatmap package in the R software.

### miRNA-related expression signature building and enrichment analysis of TMB-related miRNAs

The expression values of the above DE miRNAs for each CRC sample were extracted from the training cohort. The least absolute shrinkage and selection operator (LASSO) method, which could select optimal features from high-dimensional data [26], was used to screen the most useful predictive miRNAs for TMB level. The miRNAs, whose regression coefficients were non-zero in the LASSO regression analysis, were identified as TMB-related miRNAs. The miRNA-related expression signature was developed based on these TMB-related miRNA expression value multiplied by their corresponding LASSO regression coefficient. To further understand the biological significance and essential pathways of these TMB-related miRNAs, Gene Ontology (GO) term for biological process (BP) and Kyoto Encyclopedia of Genes and Genomes (KEGG) enrichment analysis were performed in DIANA-mirPath web tool [27]. To provide visual information, the enriched results for GO and KEGG analysis were presented as the bubble plot by the ggplot2 R package.

### Principal component analysis of DE miRNAs and TMB-related miRNAs and validation of the miRNA-related expression signature

To evaluate whether miRNAs could make a distinction between TMB^high^ samples and TMB^low^ ones, PCA was conducted based on the expression profiles of DE miRNAs and TMB-related miRNAs. Two PCA plots were displayed by the ggplot2 R package, in which the correlations among all CRC samples were converted into a two-dimensional graph. The validation cohort was then used to evaluate the robustness of this miRNA-related expression signature. First, the area under the receiver operating characteristic curve (AUC) was measured to assess the discrimination ability of this expression signature by the pROC R package [28]. Generally, AUC above 90% means that this signature is almost perfect. Then, the calibration performance of this expression signature was assessed using the “rms” package in the R software [29]. The calibration plot was presented accompanied with the unreliability test, in which *P* value > 0.05 means that this classifier calibrated perfectly with the ideal signature. Finally, we performed decision curve analysis (DCA) to assess the clinical utility of this expression signature, and net benefit at different threshold probabilities was calculated by the rmda R package [30].

### The correlation analysis between the miRNA-related expression signature and three immune checkpoints

The expression profiles of three immune checkpoints (PD-1, PD-L1, and CTLA-4) in RNA sequencing were downloaded from TCGA. Log2 (count+1) transformation was used to normalize the expression profiles of these three immune checkpoints. In the overall cohort, the expression signature value of each sample was calculated, and the correlation between the miRNA-related expression signature and three immune checkpoints was analyzed. Besides, we used the TargetScan webserver to identify which immune checkpoint could serve as a potential target gene of these TMB-related miRNAs [31].

### Statistical analysis

The clinical information between the training cohort and validation cohort was displayed as categorical data and analyzed by the *χ*^2^ test in the R software. The Wilcoxon test was used to analyze the miRNA expression levels between the TMB^high^ group and the TMB^low^ group and was applied using the R software. A *P* value < 0.05 was regarded as a statistical difference.

## Results

### Identification of DE miRNAs

**Fig. 1:**
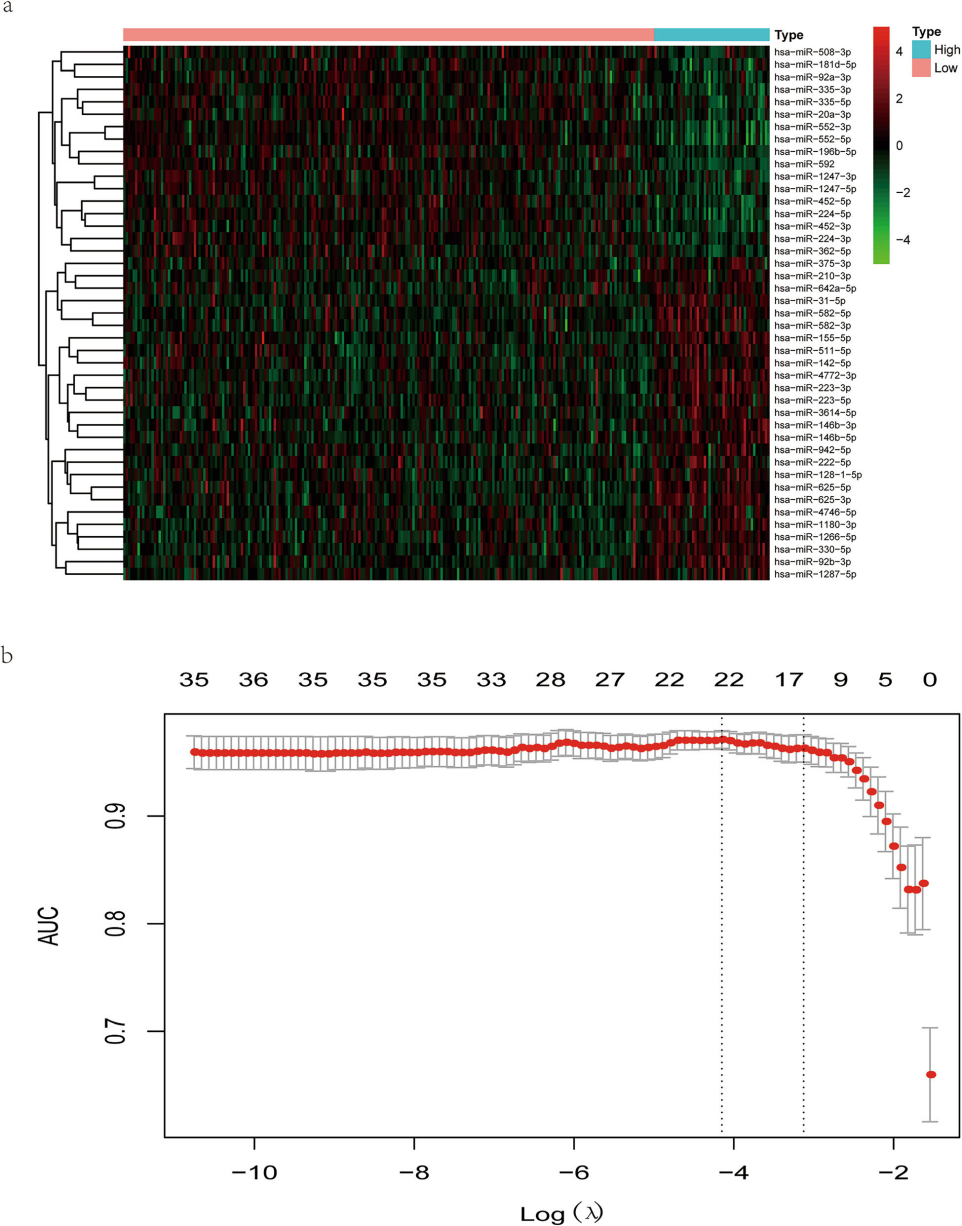
Identification of TMB-related miRNAs. **a** The heatmap of differentially expressed miRNAs (DE miRNAs). Each column represented each sample. The red dots in the heatmap represented upregulation, the green dots represented downregulation, and black dots represented miRNAs without differential expression. **b** Development of a TMB-related miRNA expression signature by the least absolute shrinkage and selection operator (LASSO) method. The optimal miRNAs with non-zero regression coefficients (*λ*) was selected by 10-fold cross-validation and “AUC” measure type

As was shown in Table 1, no statistical difference was found in baseline characteristics between the training and validation cohort, including the clinical stage and TNM stage. There were 226 TMB^low^ samples and 49 TMB^high^ samples in the training cohort, among which 43 DE miRNAs were obtained with threshold criteria of |log2 FC| > 1.5 and FDR < 0.01. To visualize the expression patterns of these DE miRNAs, a heatmap was displayed, in which 26 upregulated miRNAs and 17 downregulated miRNAs were identified (Fig. 1a).

**Table 1:** Baseline characteristics of patients in the total, training, and validation cohort

Characteristics	Total cohort (*N* = 457)	Training cohort, (*N* = 275)	Validation cohort (*N* = 182)	*P* value
**Age**				
≤ 65	205 (44.86%)	128 (46.55%)	77 (42.31%)	0.4262
> 65	252 (55.14%)	147 (53.45%)	105 (57.69%)	
**Gender**				
Female	221 (48.36%)	138 (50.18%)	83 (45.6%)	0.3882
Male	236 (51.64%)	137 (49.82%)	99 (54.4%)	
**Stage**				
Stages I–II	246 (53.83%)	144 (52.36%)	102 (56.04%)	0.3331
Stages III–IV	196 (42.89%)	124 (45.09%)	72 (39.56%)	
Not available	15 (3.28%)	7 (2.55%)	8 (4.4%)	
**T**				
T1–2	89 (19.47%)	52 (18.91%)	37 (20.33%)	0.6735
T3–4	367 (80.31%)	222 (80.73%)	145 (79.67%)	
Tis	1 (0.22%)	1 (0.36%)	0 (0%)	
**M**				
M0	326 (71.33%)	194 (70.55%)	132 (72.53%)	0.8493
M1	68 (14.88%)	43 (15.64%)	25 (13.74%)	
MX/not available	63 (13.79%)	38 (13.82%)	25 (13.74%)	
**N**				
N0	261 (57.11%)	149 (54.18%)	112 (61.54%)	0.2282
N1–2	195 (42.67%)	125 (45.45%)	70 (38.46%)	
NX	1 (0.22%)	1 (0.36%)	0 (0%)

### miRNA-related expression signature building and enrichment analysis of these TMB-related miRNAs

**Fig. 2:**
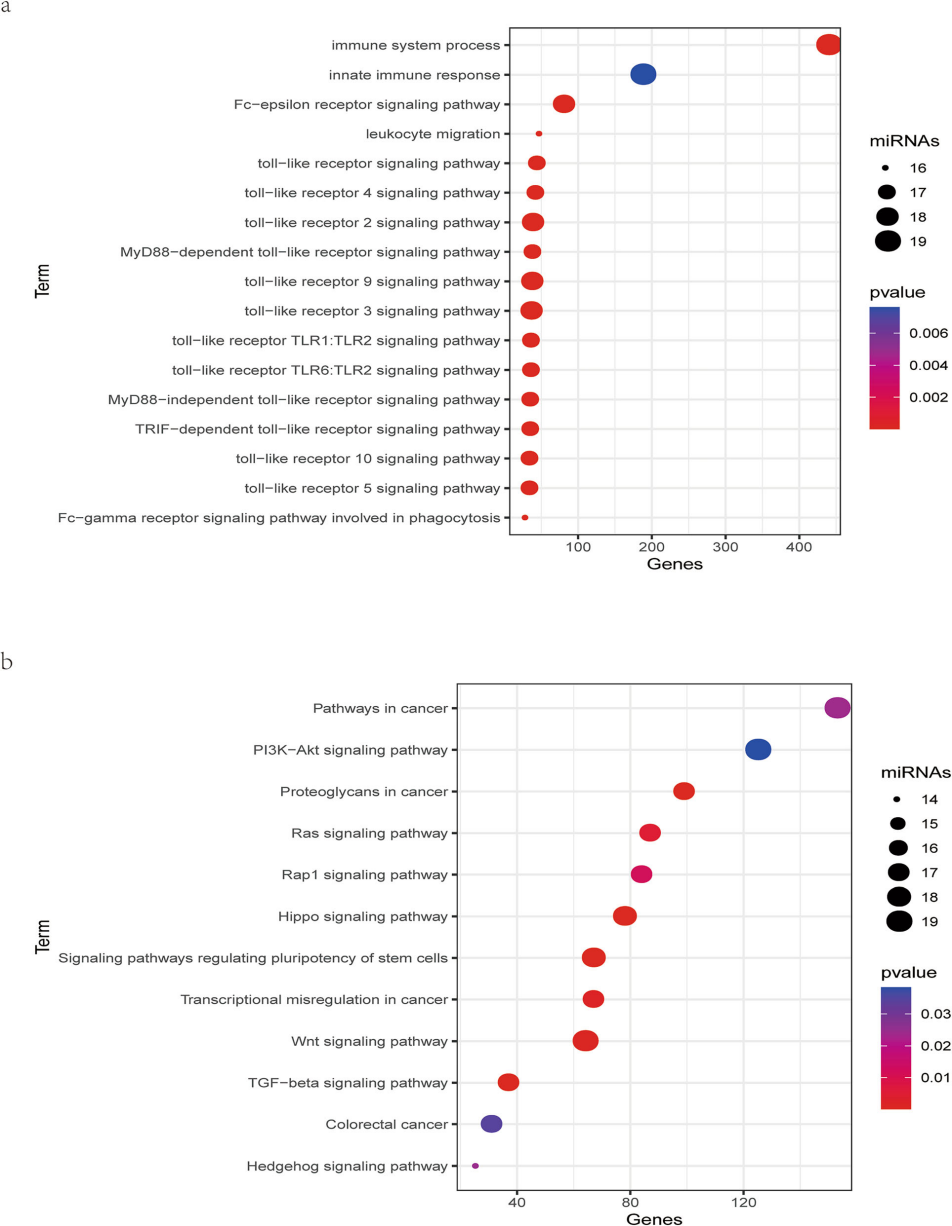
Functional enrichment analysis of TMB-related miRNAs. **a** GO term for BP of DE miRNAs. **b** KEGG pathway of DE miRNAs

Based on the expression profiles of DE miRNAs in the training cohort, we used LASSO method to identify the most useful predictive miRNAs for TMB level. 21 miRNAs, whose coefficients were non-zero under the biggest AUC, were regarded as optimal features and used to develop a miRNA-related expression signature (Fig. 1b). The formula for this miRNA-related expression signature was as follows: formula = − 5.14131584 + [hsa-miR-92b-3p expression × (0.26483929)] + [hsa-miR-942-5p × (0.43196635)] + [hsa-miR-452-5p × (− 0.29214023)] + [hsa-miR-223-3p × (0.00493161)] + [hsa-miR-1247-3p × (− 0.08271914)] + [hsa-miR-4746-5p × (0.09968608)] + [hsa-miR-592 × (− 0.23263551)] + [hsa-miR-1180-3p × (0.08740547)] + [hsa-miR-1266-5p × (0.13386031)] + [hsa-miR-155-5p × (0.32834847)] + [hsa-miR-552-3p × (− 0.06864561)] + [hsa-miR-146b-5p × (0.31452767)] + [hsa-miR-552-5p × (− 0.31677114)] + [hsa-miR-375-3p × (0.00911625)] + [hsa-miR-224-5p × (− 0.27275485)] + [hsa-miR-452-3p × (− 0.35895800)] + [hsa-miR-582-5p × (0.53131818)] + [hsa-miR-330-5p × (0.16353677)] + [hsa-miR-582-3p × (0.32472284)] + [hsa-miR-92a-3p × (− 0.46113762)] + [hsa-miR-625-3p × (0.30478831)]. As was shown in Fig. 2a, the GO enrichment terms for BP were mainly associated with immune response, such as the innate immune response, leukocyte migration, and toll-like receptor signaling pathway, and the like. Besides, the KEGG results revealed that these miRNAs were related with cancer-related pathways, such as the colorectal cancer pathway, Ras signaling pathway, TGF-beta signaling pathway, and Wnt signaling pathway (Fig. 2b).

### PCA and validation of the miRNA-related expression signature

**Fig. 3:**
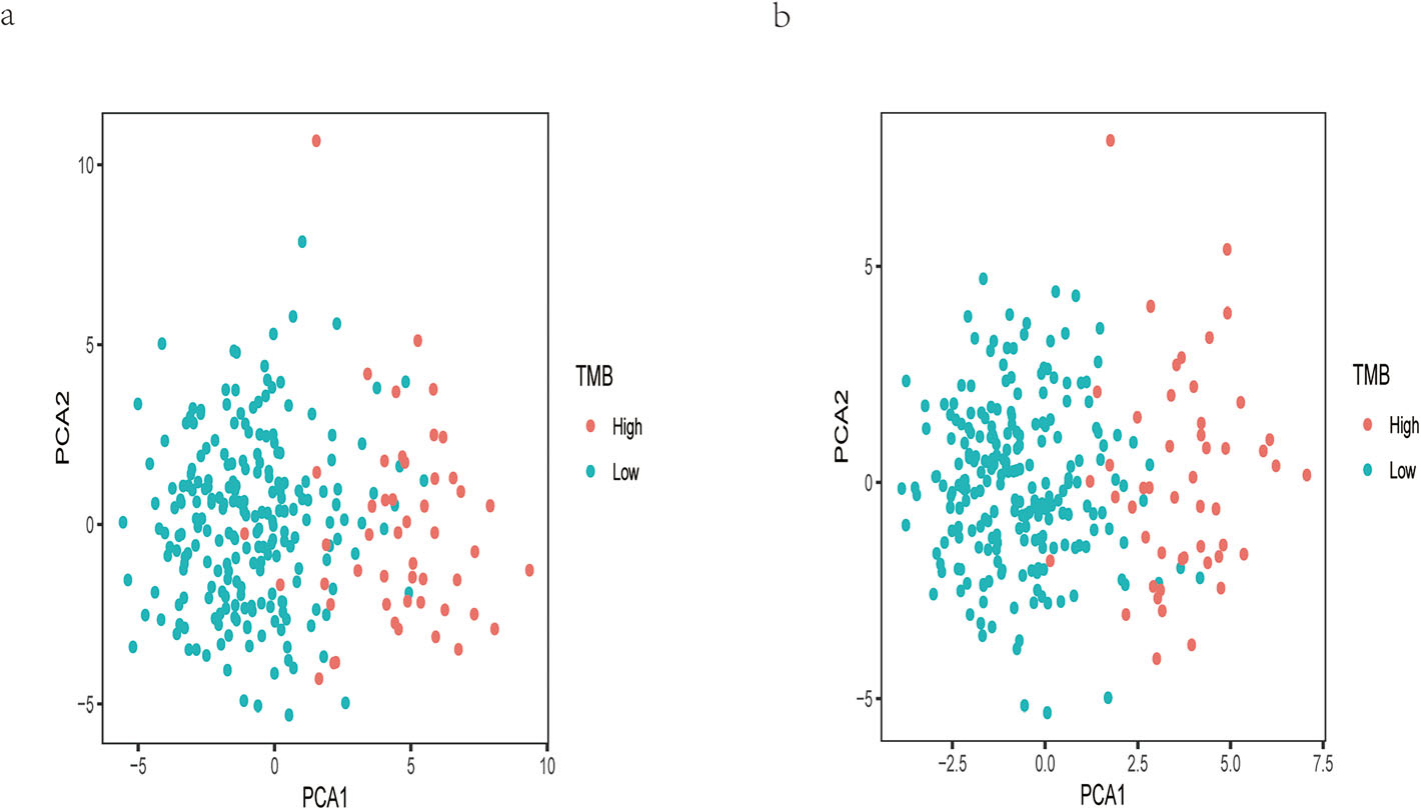
PCA of DE miRNAs and TMB-related miRNAs

**Fig. 4:**
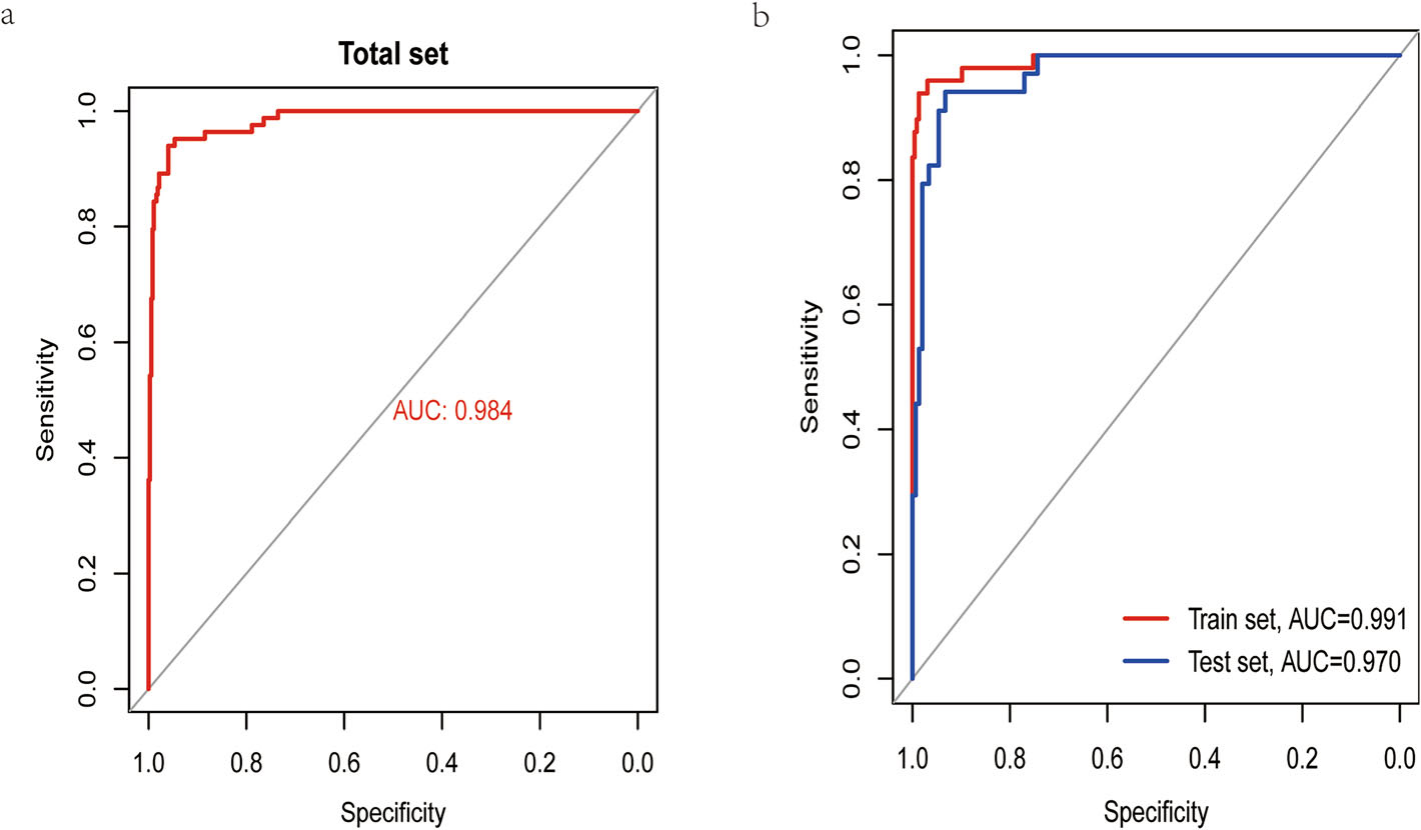
The AUC in the training, validation, and overall cohort

**Fig. 5:**
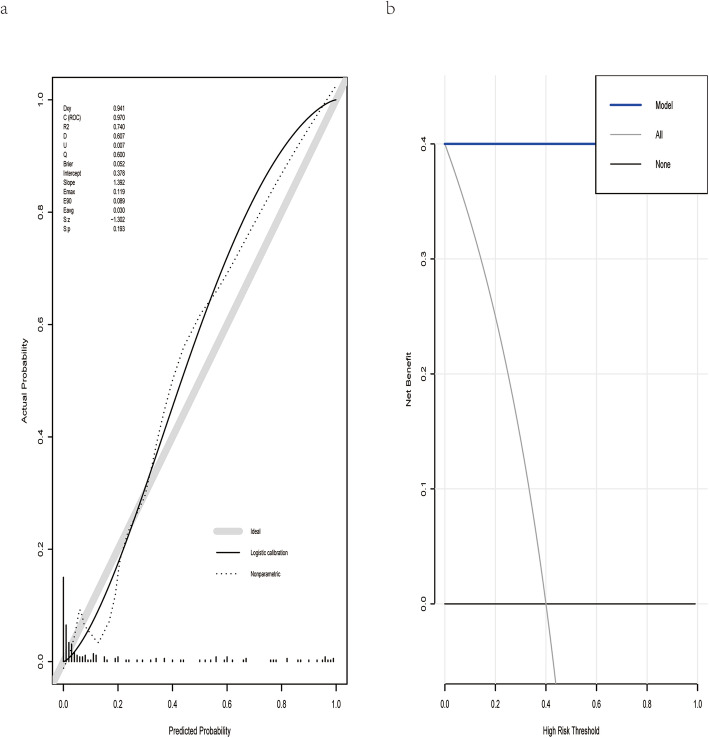
Calibration and DCA for this expression signature

As was shown in Fig. 3, the PCA results for either DE miRNAs or TMB-related miRNAs revealed that the above two types of miRNAs could obviously make a distinction between TMB^high^ samples and TMB^low^ ones. The AUC of this miRNA-related expression signature was 0.991, 0.970, and 0.984 in the training, validation, and overall cohort, respectively (Fig. 4). Besides, the specificity (SP), negative predictive value (NPV), sensitivity (SE), positive predictive value (PPV), and accuracy values were very high (Table 2), indicating that this signature could perfectly discriminate patients with different TMB levels. The calibration plot revealed this signature had no departure from perfect fit, and no statistical difference was found between this signature and ideal signature (*P* > 0.05) (Fig. 5a). DCA demonstrated that whatever the threshold probability was, this signature could produce more benefit than either treating-no-one curve or treating-everyone curve, suggesting this was a perfect signature (Fig. 5b).

**Table 2:** Predictive performance of miRNA-based expression signature for tumor mutational burden in CRC

Cohort	SE	SP	PPV	NPV	Accuracy	AUC
Total	0.8434	0.9866	0.9333	0.966	0.9606	0.9839
Training	0.8776	0.9956	0.9773	0.974	0.9745	0.9913
Validation	0.7941	0.973	0.871	0.9536	0.9396	0.9704

*SE* sensitivity, *SP* specificity, *PPV* positive predictive value, *NPV* negative predictive value, *AUC* area under the receiver operating characteristic curve

### Positive correlation between the expression signature and three immune checkpoints

**Fig. 6:**
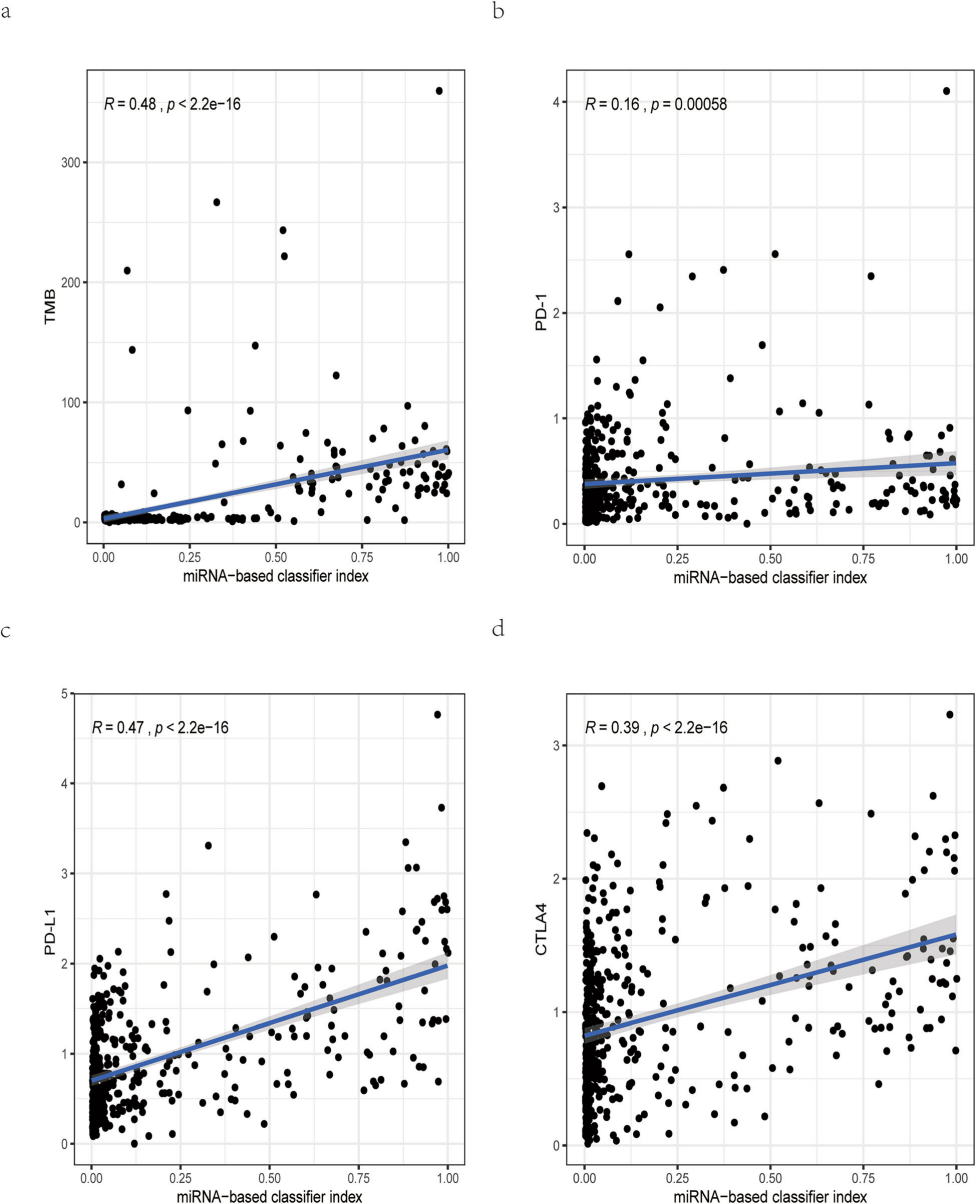
The correlation analysis between this miRNA-related expression signature and three immune checkpoints. **a** This expression signature is moderately correlated with TMB. **b** This expression signature is low correlated with PD-1. **c**, **d** This expression signature is moderately correlated with PD-L1 and CTLA-4

**Fig. 7:**
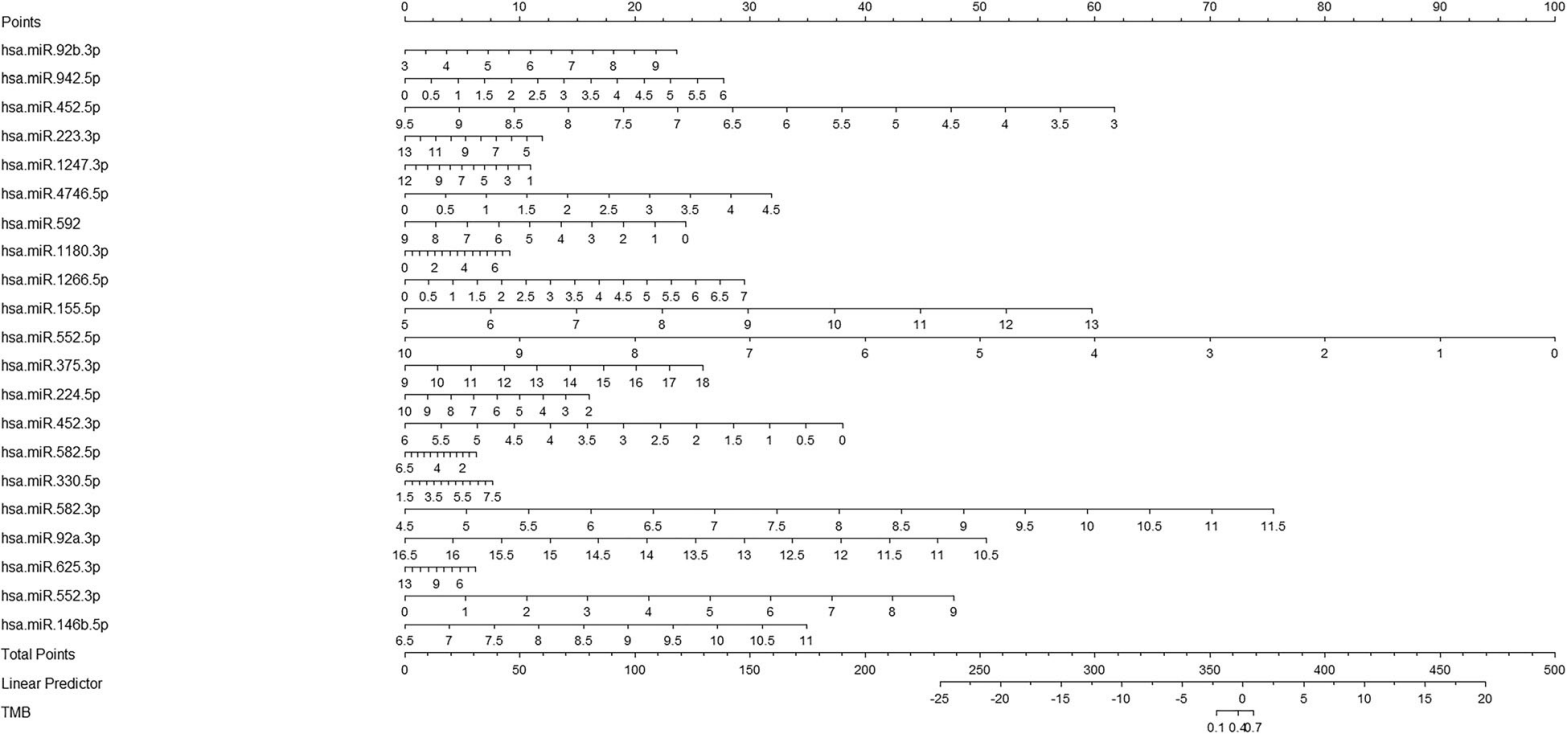
Nomogram for this miRNA-related expression signature. This nomogram is developed based on TMB related miRNAs in the training cohort

Not surprisingly, this miRNA-related expression signature, which could assess TMB level for CRC patients, showed a median positive correlation with TMB (*R* = 0.48, *P* < 2.2e−16). Besides, for three immune checkpoints, this signature was moderately correlated with PD-L1 (*R* = 0.47, *P* < 2.2e−16) and CTLA4 (*R* = 0.39, *P* < 2.2e−16), and not significantly correlated with PD-1 (R = 0.16, *P* = 0.00058) (Fig. 6). Interestingly, based on the results of miRNA-mRNA in TargetScan, PD-L1, CTLA4, and PD-1 were targeted by 2 (hsa-miR-155-5p, hsa-miR-552-5p), 3 (hsa-miR-942-5p, hsa-miR-155-5p, hsa-miR-582-3p), and 4 (hsa-miR-223-3p, hsa-miR-1247-3p, hsa-miR-592, hsa-miR-552-5p) miRNAs, respectively. Finally, in order to help clinicians more conveniently apply this miRNA-related expression signature, we transformed this signature into a visual nomogram (Fig. 7).

## Discussion

Though PD-L1 expression and MSI-H are established biomarkers for predicting response to ICIs in CRC [8, 9], the response rate to ICIs among CRC patients with high PD-L1 expression or MSI-H is variable. TMB is an emerging independent biomarker for predicting response to ICIs in CRC [11]. However, the current TMB assessment has not been standardized, affecting its wide application in clinical practice [14]. Considering that miRNAs were found involved in the regulation of anti-cancer immune response [21, 32–36], we developed a miRNA-related expression signature, which could be a good addition to PD-L1 or MSI-H and help identify CRC patients with different TMB levels. The relationship between TMB and miRNA expression profile was not analyzed in previous studies. In this study, DE miRNAs between the TMB^high^ group and the TMB^low^ group were identified and then incorporated into the LASSO regression analysis. Some miRNAs with non-zero coefficients in the LASSO method as TMB-related miRNAs were used to develop a miRNA-related expression signature. In the validation cohort, this signature demonstrated satisfactory discrimination, great calibration, and more benefit in clinical utility, indicating that this signature was a robust classifier for TMB levels. In particular, the SP, NPV, SE, and PPV of this signature were very high, suggesting that this signature had great recognition ability for TMB^low^ samples and TMB^high^ samples, respectively.

Previous researches revealed that TMB^high^ patients could produce more somatic mutations and neoantigen loads which could activate anti-cancer immune response [10]. Interestingly, functional enrichment analysis for TMB-related miRNAs revealed that these miRNAs were mainly involved in biological processes related with immune response and signaling pathways associated with cancer. Besides, the miRNA-related expression signature showed a median positive correlation with TMB, indicating that this miRNA-related expression signature predicted the TMB level from a biological perspective of the anti-cancer immune response.

Several studies have revealed that miRNAs could decrease PD-L1 expression by binding to 3′ UTR of PD-L1, suggesting that miRNAs were negatively related with PD-L1 expression [21, 33, 34]. However, in our research, this miRNA expression signature was positively related with three immune checkpoints, especially for PD-L1 expression. Thus, future researches are required to explore the underlying mechanism between these TMB-related miRNAs and PD-L1 expression.

Some limitations should be acknowledged in our research. First, the cutoff point to separate samples into the TMB^high^ group and the TMB^low^ group may vary for different TMB detection methods. Second, a larger independent cohort is required to validate the robustness of this miRNA-related expression signature. Finally, the potential mechanisms between TMB-related miRNAs and CRC immune response are not elucidated. Future studies are required to explore the underlying mechanisms.

## Conclusion

In summary, we developed a miRNA-related expression signature, which could stratify CRC patients with different TMB levels and further help clinicians evaluate the efficacy of ICIs. This signature was well-validated with reference to its discrimination, calibration, and DCA.

## Data Availability

All the data and materials are available.

## Abbreviations

AUC: Area under the receiver operating characteristic curve
BP: Biological process
CRC: Colorectal cancer
CTLA-4: Cytotoxic T-lymphocyte antigen 4
DCA: Decision curve analysis
DE miRNAs: Differentially expressed miRNAs
FC: Fold change
FDR: False discovery rate
GO: Gene Oncology
ICIs: Immune checkpoint inhibitors
KEGG: Kyoto Encyclopedia of Genes and Genomes
LASSO: Least absolute shrinkage and selection operator
miRNAs: MicroRNAs
MSI: Microsatellite instability
MSI-H: High microsatellite instability
NPV: Negative predictive value
PCA: Principal component analysis
PD-1: Programmed death protein 1
PD-L1: Programmed death receptor ligand 1
PPV: Positive predictive value
SE: Sensitivity
SP: Specificity
TMB: Tumor mutational burden
UTR: Untranslated region
>hsa-miR-92b-3p MIMAT0003218: .
UAUUGCACUCGUCCCGGCCUCC: .
>hsa-miR-942-5p MIMAT0004985: .
UCUUCUCUGUUUUGGCCAUGUG: .
>hsa-miR-452-5p MIMAT0001635: .
AACUGUUUGCAGAGGAAACUGA: .
>hsa-miR-223-3p MIMAT0000280: .
UGUCAGUUUGUCAAAUACCCCA: .
>hsa-miR-1247-3p MIMAT0022721: .
CCCCGGGAACGUCGAGACUGGAGC: .
>hsa-miR-4746-5p MIMAT0019880: .
CCGGUCCCAGGAGAACCUGCAGA: .
>hsa-miR-592 MIMAT0003260: .
UUGUGUCAAUAUGCGAUGAUGU: .
>hsa-miR-1180-3p MIMAT0005825: .
UUUCCGGCUCGCGUGGGUGUGU: .
>hsa-miR-1266-5p MIMAT0005920: .
CCUCAGGGCUGUAGAACAGGGCU: .
>hsa-miR-155-5p MIMAT0000646: .
UUAAUGCUAAUCGUGAUAGGGGUU: .
>hsa-miR-552-3p MIMAT0003215: .
AACAGGUGACUGGUUAGACAA: .
>hsa-miR-146b-5p MIMAT0002809: .
UGAGAACUGAAUUCCAUAGGCUG: .
>hsa-miR-552-5p MIMAT0026615: .
GUUUAACCUUUUGCCUGUUGG: .
>hsa-miR-375-3p MIMAT0000728: .
UUUGUUCGUUCGGCUCGCGUGA: .
>hsa-miR-224-5p MIMAT0000281: .
UCAAGUCACUAGUGGUUCCGUUUAG: .
>hsa-miR-452-3p MIMAT0001636: .
CUCAUCUGCAAAGAAGUAAGUG: .
>hsa-miR-582-5p MIMAT0003247: .
UUACAGUUGUUCAACCAGUUACU: .
>hsa-miR-330-5p MIMAT0004693: .
UCUCUGGGCCUGUGUCUUAGGC: .
>hsa-miR-582-3p MIMAT0004797: .
UAACUGGUUGAACAACUGAACC: .
>hsa-miR-92a-3p MIMAT0000092: .
UAUUGCACUUGUCCCGGCCUGU: .
>hsa-miR-625-3p MIMAT0004808: .
GACUAUAGAACUUUCCCCCUCA: .
